# Information processing in computer-assisted interventions: 4th international conference, 2013

**DOI:** 10.1007/s11548-014-1117-6

**Published:** 2014-09-20

**Authors:** Dean Barratt, Pierre Jannin, Gabor Fichtinger, Stephane Cotin

**Affiliations:** 1 UCL Centre for Medical Image Computing, Department of Medical Physics and Biomedical Engineering, University College London, Floor 2, Malet Place Engineering Building, Gower Street, London, WC1E 6BT UK; 2 LTSI, Inserm UMR 1099, Faculté de Médecine, Université de Rennes 1, 2, Avenue du Pr. Léon Bernard, 35043 Rennes Cedex, France; 3 Laboratory for Percutaneous Surgery, School of Computing, Queen’s University, 557 Goodwin Hall, Kingston, ON K7L 2N8 Canada; 4 Equipe Projet SHACRA, INRIA, 40 Avenue Halley, 59650 Villeneuve d’Ascq, France

Information processing is a fundamental and critical task in computer-aided surgery and interventional radiology. Within surgical and radiological procedures, the primary role of assistive computer systems is to present clinically salient information to facilitate significant improvements in instrument localisation accuracy, patient safety, efficacy, clinical outcomes, and patient recovery times (for example, by enabling less-invasive techniques). Further important potential benefits are the reduction in the technical skill required to perform a procedure and an elevated confidence that the procedure has been performed successfully, which is associated with improved outcomes in clinical training. De-skilling and improved clinical confidence can also lead to significant reductions in the time required to perform some procedures, thereby reducing per-procedure costs. However, in practice, there are many examples where this advantage is compromised by the overhead of introducing new complex medical technology and/or non-standard workflows. The capital costs of advanced computer-assisted equipment can also be high, particularly for those that incorporate complex, state-of-the-art technologies.

Continued advances in computer hardware and computational power now mean that very large quantities of data are typically acquired and processed at all stages of the clinical pathway, from disease diagnosis and localisation, planning and subsequent execution of interventional procedures, through to post-procedural analysis and patient follow-up. Much of these data are produced by imaging devices, but data also originate from a number of other sources including (non-imaging) sensing devices, such as intraoperative navigation devices, and traditional clinical data, such as pathology results, patient risk, patient history, and many more.

Processing and analysing medical data to extract and present clinically important information in different interventional scenarios remains highly challenging from a number of perspectives: firstly, automatic and robust extraction of the most relevant information is not always easy given that noise, artefacts, and complicating temporal changes (due, for example, to soft-tissue motion and dynamic physiological processes) are all commonplace in clinical data. Consequently, computer-aided interventional systems often require complex computer algorithms. Combining data/information in a clinically optimal and informative way is often challenging and typically involves advanced computational methods for registering and fusing spatial and temporal data from different sources. Such methods increasingly rely on complex, patient-specific computer modelling of human anatomy and physical and physiological processes. Integrating these technologies within computer-aided devices in a way that they function reliably and robustly within safety critical clinical environments is difficult, time-consuming, and resource-intensive from an engineering perspective. Clinical validation and evaluation, which are of critical importance, are also typically challenging, both methodologically and practically. Finally, even the most sophisticated systems can require input from a human operator, and all provide information that informs or guides a procedure in some way. Therefore, human–computer interaction and workflow analysis are extremely important considerations in the design of computer-assisted systems that are not trivial for complex surgical tasks and demand new methodologies.

Addressing these problems is an inherently interdisciplinary research activity that requires in-depth knowledge of medical imaging and sensing, advanced computational techniques for analysing and processing clinical data, systems and software engineering, as well as clinical applications, including clinical user requirements, surgical workflows, and the clinical context of specific diseases and surgical procedures. Intellectually, research in this area involves the analysis of healthcare problems from both an engineering and clinical perspective, and the design, construction, and validation of complex computer-based systems that can be used effectively by clinicians in time-critical surgical environments. At the core of research in this area is an active and rapidly growing community of specialists in information processing for computer-assisted interventions. The growth of this community is driven by the ever-increasing clinical and public demand for technologies that address the world’s most pressing healthcare needs. As an academic discipline, the field therefore has very strong potential for growth and healthcare impact.

The International Conference on Information Processing in Computer-Assisted Interventions (IPCAI) is an international forum for this community, organised by leaders in the field. The conference provides a venue for the presentation of novel technical innovations, systems development, and clinical studies related to computer-assisted interventions, with the aim of becoming the premier international meeting for discussing technical concepts; clinical needs and applications; and hardware, software, and systems and their validation.

This special issue of IJCARS is the first containing original papers presented at the IPCAI conference. All presenting authors were invited to submit a revised and extended version of their short paper originally presented at the conference, including more methodological detail, further experimental results, and more comprehensive discussion than the original conference proceedings [1]. All submissions were peer-reviewed by the IPCAI programme chairs and other invited expert reviewers. The accepted papers represent a small selection of the contributions presented at the 4th IPCAI conference, held in Heidelberg, Germany, in 2013. They do, however, reflect the breadth of activity presented at the conference, in IJCARS, and the field in general, covering the key topics of 3D tracking for instrument navigation [2], intraoperative image segmentation for online radiation dosimetry [3], image-based anatomical models for catheter placement [4], and task and crisis analysis during surgical training through the combination of virtual reality and monitoring human multisensory responses to provide an immersive environment [5].

In [2], a new mobile electromagnetic (EM) field generator, part of a small EM 3D position tracking system, is attached to an ultrasound transducer as part of 3D needle guidance system. This innovation addresses the well-known problems of position measurement errors from EM field distortion due to metal in the environment, and the difficulties associated with optimal placement of the field generator with respect to the tracker sensor in conventional EM tracking devices, to maximise instrument localisation accuracy within a region of interest.

In [3], the authors describe an automatic method for detecting and localising brachytherapy seeds implanted into the prostate from C-arm X-ray images. This technique overcomes the issues of extended procedure times and operator error associated with manual identification of seeds, which limit the practical feasibility of online, dynamic radiation therapy planning in this application.

Reinersten et al. [4] compare an approach to placing external ventricular drains into the brain during cerebrospinal diversion procedures that employs an ‘average’ image-based anatomical brain model with a conventional freehand approach. They conclude that the image-informed approach, whilst not patient-specific, resulted in a significant improvement in catheter placement accuracy compared with the standard surgical procedure.

Finally, Wucherer et al. [5] present a very interesting account of the design of and initial experience with a prototype immersive surgical training simulator that combines a virtual reality procedural simulator with a computerised mannequin to devise novel training setups, and a quantitative evaluation surgical workflow and crisis simulation using this new technology.
